# Prediction model for early graft failure after liver transplantation using aspartate aminotransferase, total bilirubin and coagulation factor

**DOI:** 10.1038/s41598-021-92298-6

**Published:** 2021-06-18

**Authors:** Jinsoo Rhu, Jong Man Kim, Kyunga Kim, Heejin Yoo, Gyu-Seong Choi, Jae-Won Joh

**Affiliations:** 1 Department of Surgery, Samsung Medical Center, Sungkyunkwan University School of Medicine, 50 Irwon-dong, Gangnam-gu, Seoul, 135-710 Korea; 2 Statistics and Data Center, Research Institute for Future Medicine, Samsung Medical Center, Seoul, Korea

**Keywords:** Gastroenterology, Medical research

## Abstract

This study was designed to build models predicting early graft failure after liver transplantation. Cox regression model for predicting early graft failure after liver transplantation using post-transplantation aspartate aminotransferase, total bilirubin, and international normalized ratio of prothrombin time was constructed based on data from both living donor (n = 1153) and deceased donor (n = 359) liver transplantation performed during 2004 to 2018. The model was compared with Model for Early Allograft Function Scoring (MEAF) and early allograft dysfunction (EAD) with their C-index and time-dependent area-under-curve (AUC). The C-index of the model for living donor (0.73, CI = 0.67–0.79) was significantly higher compared to those of both MEAF (0.69, P = 0.03) and EAD (0.66, P = 0.001) while C-index for deceased donor (0.74, CI = 0.65–0.83) was only significantly higher compared to C-index of EAD. (0.66, P = 0.002) Time-dependent AUC at 2 weeks of living donor (0.96, CI = 0.91–1.00) and deceased donor (0.98, CI = 0.96–1.00) were significantly higher compared to those of EAD. (both 0.83, P < 0.001 for living donor and deceased donor) Time-dependent AUC at 4 weeks of living donor (0.93, CI = 0.86–0.99) was significantly higher compared to those of both MEAF (0.87, P = 0.02) and EAD. (0.84, P = 0.02) Time-dependent AUC at 4 weeks of deceased donor (0.94, CI = 0.89–1.00) was significantly higher compared to both MEAF (0.82, P = 0.02) and EAD. (0.81, P < 0.001). The prediction model for early graft failure after liver transplantation showed high predictability and validity with higher predictability compared to traditional models for both living donor and deceased donor liver transplantation.

## Introduction

Liver transplantation (LT) is a life-saving procedure for patients with acute or chronic liver failure and malignancy such as hepatocellular carcinoma. However, due to organ shortage, LT can only be performed in a limited number of patients. Nevertheless, LT is not always successful, and 2.7 to 6.9% of liver grafts develop graft dysfunction^1–3^. Dysfunction of the graft, whether the cause is primary or secondary, can lead to death or need for additional liver transplantation. Currently in the United States, the Organ Procurement and Transplant Network (OPTN) set an urgent listing criteria for primary nonfunction of a transplanted liver within 7 days of implantation^4^. The recipients should be in an anhepatic phase or should have aspartate aminotransferase (AST) ≥ 3000 U/L and one or both of the following: international normalized ratio (INR) of prothrombin time ≥ 2.5, or acidosis, defined as arterial pH ≤ 7.30 or venous pH ≤ 7.25 and/or lactate ≥ 4 mmol/L. However, the criteria of OPTN seem restrictive, and many patients who do not fulfill the criteria experience graft failure.

To overcome these conditions, many clinicians have reported on graft dysfunction which can role as an indicator for future graft failure^1–3,5–10^. However, the criteria of graft dysfunction vary significantly among studies, and markers for graft dysfunction vary widely, including AST^2,3,5,8^, alanine aminotransferase (ALT)^1,8,9^, prothrombin time^2,3,8^, total bilirubin (TB)^11^, acidosis^12^, and ammonia^3^. The time points at which those laboratory values are measured varies significantly among studies but are usually within the first week after LT^2,3,9,13^. One of the criteria suggested was early allograft dysfunction (EAD) criteria suggested by Olthoff et al., and modeling early allograft function (MEAF) score^14,15^. Nevertheless, these models still have limitations to be chosen as indicator for retransplantation of the LT recipient.

Therefore, we designed this study to build a prediction model for predicting early graft failure of which endpoint has been defined as retransplantation of the liver or death due to graft dysfunction with three goals. First, to design both living donor liver transplantation (LDLT) and deceased donor liver transplantation (DDLT) models to predict graft survival using common laboratory tests. The second goal is to compare the predictability with other known models. The third goal is to internally validate the prediction model. The calculating model designed for predicting early graft failure will be abbreviated as ABC model by including AST, TB, and INR of prothrombin time which is a coagulation factor.

## Methods

### Patients

The study population consisted of adult patients who underwent LT in Samsung Medical Center during the period of 2004 to 2018. Pediatric LTs were excluded, while both living donor and deceased donor LTs of adult recipients were included. No organs from executed prisoners were used.

### Data collection

Patient data of demographics, LT surgery, and post-transplantation course including laboratory values of AST, TB, and INR were collected from the date of transplantation to the 7th day post-transplantation.

### Graft failure

Graft failure was defined as failure of the liver allograft, either primary or secondary, due to complications that required re-LT or resulted in death of the recipient. The date of graft failure was defined as the date of re-LT or death. Deaths from causes other than liver failure were not defined as graft failure.

### Post-transplantation laboratory values

AST, TB, and INR were used to predict graft survival. Laboratory values during the first week were used. Since laboratory values during the early post-transplantation period can be influenced by pre-transplantation conditions, some modifications were made. TB and INR levels from the day of LT to post-LT day 2 were not used for the prediction model since TB and INR gradually decrease along the post-LT course even in successful LT. Therefore, for the prediction model, maximum level of AST during the first week (AST_max7_), maximum level of TB from days 3 to 7 post-LT (TB_max3–7_), and maximum INR from days 3 to 7 post-LT (INR_max3–7_) were used to predict graft survival.

### Statistical analysis

The prediction models were built using variables that are clinically familiar and relevant. Two models each for LDLT and DDLT were constructed. After building the models, the two models were compared to MEAF score and EAD criteria by comparing C-index and time dependent area-under-the-curve (AUC) at 2 weeks and 4 weeks^14,15^. MEAF score was calculated based on the previous study reported by Pareja et al.^15^ The comparing process was performed using R packages 'compareC' and 'timeROC'. Validation process for the chosen modeling process was performed. Internal validation using 20-time repeated fivefold cross-validations were performed using R package 'survAUC' to calculate the C-statistic and AUC estimator proposed by Uno et al.^16^ Calibration plot was drawn to validate the models through 1000 bootstrap resamples of the same size as the original data. Decision curve analysis to evaluate the clinical usefulness of the models was performed by drawing a decision curve computing the net benefit, and the range of positive net benefit was analyzed.

Statistical analyses were performed using SPSS 20.0 (IBM, Chicago, IL, USA), SAS v9.4 (SAS Institute Inc, Cary, NC, USA), and R 3.6.1 (R Foundation for Statistical Computing, Vienna, Austria) using packages 'rmda' for decision curve analysis and 'rms' for drawing a calibration plot.

### Ethical approval

This study was approved by the Institutional Review Board (IRB) of Samsung Medical Center (IRB No. 2020-02-013).

### Informed consent

The need for informed consent was waived by the IRB of Samsung Medical Center due to the retrospective nature of this study. Investigational methods used in this study were implemented in accordance with the relevant guidelines and regulations of the IRB.

## Results

### Characteristics of the patient group

Table 1 shows the summary of baseline characteristics and post-LT courses of the patient group. A total of 1512 LTs, 1153 LDLTs and 359 DDLTs were included to the study. Most of the recipients were male patients (78.0%, n = 899 in LDLT and 67.1%, n = 241 in DDLT) with mean age around 52. (52.8 ± 8.5 years in LDLT and 51.7 ± 10.4 years in DDLT) While mean donor age was 32.7 ± 11.6 years in LDLT, mean donor age of DDLT was 46.9 ± 16.3 years in DDLT. While only 0.9% (n = 10) of LDLTs were re-LT cases, 13.7% (n = 49) of DDLT cases were re-LT cases. While 71.8% (n = 827) of LDLT patients were hepatitis B-related, only 58.3% (n = 208) of DDLTs were hepatitis B-related. While 10.0% (n = 115) of LDLTs were alcohol-related, 26.1% (n = 93) of DDLTs were alcohol-related. While more than half (n = 670, 58.1%) of LDLT patients were HCC patients, only 38.4% (n = 138) of DDLTs were HCC patients. Number of ABO incompatible LDLTs were 149 (n = 12.9%). Median warm ischemic times and cold ischemic times were 30 min (IQR 18–40) and 83 (IQR 65–102) for LDLT and 34 min (IQR 25–44) and 273 min (IQR 210.5–356) for DDLT. Median MELD scores were 15 (IQR 10–23) for LDLT and 30 (IQR 18–39) for DDLT. Mean MEAF score was 5.77 ± 1.66 in the LDLT cases compared to 7.12 ± 1.58 in DDLT cases. While 21.0% (n = 242) of LDLT cases met the EAD criteria, 35.4% (n = 127) of DDLT cases met the EAD criteria.

**Table 1:** Baseline characteristics and postoperative graft failures of adult liver transplantation patients.

Variables	Living donor (N = 1153)	Deceased donor (N = 359)
Recipient sex (male/female)	899/254 (78.0%)	241/118 (67.1%)
Recipient mean age (years)	52.8 ± 8.5	51.7 ± 10.4
Donor sex (male/female)	746/407 (64.7%)	236/123 (65.7%)
Donor mean age (years)	32.7 ± 11.6	46.9 ± 16.3
Re-transplantation	10 (0.9%)	49 (13.7%)
Etiology		
Hepatitis B virus	827 (71.8%)	208 (58.4%)
Hepatitis C virus	67 (5.8%)	7 (2.0%)
Alcohol	115 (10.0%)	93 (26.1%)
Others	143 (12.4%)	48 (13.5%)
Hepatocellular carcinoma	670 (58.1%)	138 (38.4%)
ABO incompatible	149 (12.9%)	–
Warm ischemic time, median (minutes)	30 (18–40)	34 (25–44)
Cold ischemic time, median (minutes)	83 (65–102)	273 (201.5–356)
Macrosteatosis, median (%)	5 (0–5)	5 (5–10)
Microsteatosis, median (%)	5 (0–10)	5 (5–10)
Recipient Child-Turcotte-Pugh score, median	8 (6–11)	11 (9–12)
Recipient MELD score, median	15 (10–23)	30 (18–39)
Graft weight (g)	676.1 ± 196.5	1173.7 ± 624.8
Graft-recipient weight ratio	1.00 ± 0.34	1.74 ± 1.02
Graft failure	85 (7.4%)	46 (12.8%)
Within 2 weeks	16 (1.4%)	10 (2.8%)
Within 4 weeks	26 (2.3%)	16 (4.5%)
Within 2 months	34 (2.9%)	21 (5.8%)
Median follow up days	1740 (541–3370)	1112 (274–2489)
Median AST (U/L) on the day of LT	247 (167–364)	88 (54–346)
1st post-LT day	286 (196–453)	766.5 (443–996)
2nd post-LT day	179 (95–316)	471 (214–868)
3rd post-LT day	92 (74–168)	230 (86–582)
4th post-LT day	69 (50–91)	88 (56–252)
5th post-LT day	55 (40–78)	62 (38–94)
6th post-LT day	50 (37–72)	43 (30–75)
7th post-LT day	47 (34–69)	39 (25–61)
Median TB (mg/dL) on the day of LT	4.2 (2.7–7.1)	14.1 (5.1–31.9)
1st post-LT day	4.2 (2.4–7.2)	8.15 (5.1–9.8)
2nd post-LT day	2.6 (1.5–4.8)	6.1 (3–9.5)
3rd post-LT day	2.3 (1.5–4.6)	5.1 (2.5–9.4)
4th post-LT day	2.2 (1.4–4.5)	4.5 (2.2–8.9)
5th post-LT day	2.1 (1.4–4.4)	4.2 (2–7.6)
6th post-LT day	2 (1.3–4.1)	3.7 (1.8–6.6)
7th post-LT day	2.1 (1.3–4.2)	3.5 (1.7–6.3)
Median PT/INR on the day of LT	3.38 (2.57–4.48)	3.63 (2.77–4.84)
1st post-LT day	2.62 (2.22–3.20)	3.26 (2.32–4.30)
2nd post-LT day	2.02 (1.74–2.34)	1.57 (1.38–2.05)
3rd post-LT day	1.58 (1.42–1.80)	1.42 (1.28–1.71)
4th post-LT day	1.47 (1.34–1.66)	1.38 (1.26–1.58)
5th post-LT day	1.40 (1.27–1.58)	1.35 (1.22–1.51)
6th post-LT day	1.36 (1.24–1.54)	1.32 (1.19–1.46)
7th post-LT day	1.31 (1.19–1.46)	1.26 (1.16–1.41)
Mean MEAF score	5.77 ± 1.66	7.12 ± 1.58
Early allograft dysfunction criteria (one of more of the following)	242 (21.0%)	127 (35.4%)
AST or ALT ≥ 2000 U/L within the first 7 days	58 (5.0%)	97 (27.0%)
TB on 7th post-LT day ≥ 10 mg/dL	82 (7.1%)	44 (12.3%)
PT/INR on 7th post-LT day ≥ 1.60	150 (13.0%)	40 (11.1%)

*MEAF* model for early allograft function, *INR* international normalized ratio, *TB* total bilirubin, *AST* aspartate aminotransferase, *ALT* alanine aminotransferase.

### Prediction model using multivariable Cox regression

To build the best model for prediction, laboratory values were analyzed using univariable and multivariable models. Log_2_-transformation was performed to increase the predictability by changing the variable to a normal distribution.

Table 2 summarizes the results of the Cox regression models for LDLT. MEAF score as a continuous variable was significantly related to graft survival (HR = 1.56, CI = 1.35–1.80, P < 0.001). EAD criteria as a binary variable was significantly related to graft survival (HR = 3.28, CI = 2.14–5.03, P < 0.001). In the univariate analysis, log_2_-transformed AST_max7_ (HR = 1.87, CI = 1.60–2.18, P < 0.001), log_2_-transformed TB_max3–7_ (HR = 1.62, CI = 1.40–1.89, P < 0.001) and log_2_-transformed INR_max3–7_ (HR = 3.99, CI = 2.99–5.32, P < 0.001) were related to graft survival. The ABC model for LDLT were constructed using three variables; log_2_-transformed AST_max7_ (HR = 1.52, CI = 1.29–1.80, P < 0.001), log_2_-transformed TB_max3–7_ (HR = 1.44, CI = 1.23–1.70, P < 0.001) and log_2_-transformed INR_max3–7_ (HR = 3.29, CI = 2.20–4.90, P < 0.001).

**Table 2:** Comparisons of C-index, time-dependent AUC at 2 weeks, and time-dependent AUC at 4 weeks between Cox proportional hazard regression models using MEAF score, EAD criteria and newly developed multivariable model for predicting graft survival of recipients who underwent living donor liver transplantation.

Models	Variables	HR	95% CI	P	C-index	95% CI	P value vs. ABC model	Time-dependent AUC at 2 weeks	95% CI	P value vs. ABC model	Time-dependent AUC at 4 weeks	95% CI	P value vs. ABC model
MEAF score		1.56	1.35–1.80	< 0.001	0.69	0.63–0.76	0.03	0.90	0.81–0.99	0.09	0.87	0.78–0.95	0.02
EAD criteria		3.28	2.14–5.04	< 0.001	0.64	0.59–0.83	0.001	0.83	0.75–0.92	< 0.001	0.84	0.78–0.91	0.02
Univariable	Log_2_(AST_max7_)	1.87	1.60–2.18	< 0.001									
	Log_2_(TB_max3–7_)	1.62	1.40–1.89	< 0.001									
	Log_2_(INR_max3–7_)	3.99	2.99–5.32	< 0.001									
Multivariable ABC model	Log_2_(AST_max7_)	1.52	1.29–1.80	< 0.001									
	Log_2_(TB_max3–7_)	1.44	1.23–1.70	< 0.001	0.73	0.67–0.79		0.96	0.91–1.00		0.93	0.86–0.99	
	Log_2_(INR_max3–7_)	3.29	2.20–4.90	< 0.001									

*HR* hazard ratio, *AUC* area under the curve, *CI* confidence interval, *MEAF* modeling early allograft function, *EAD* early allograft dysfunction, *AST* aspartate aminotransferase, *TB* total bilirubin, *INR* international normalized ratio.

**Figure 1:**
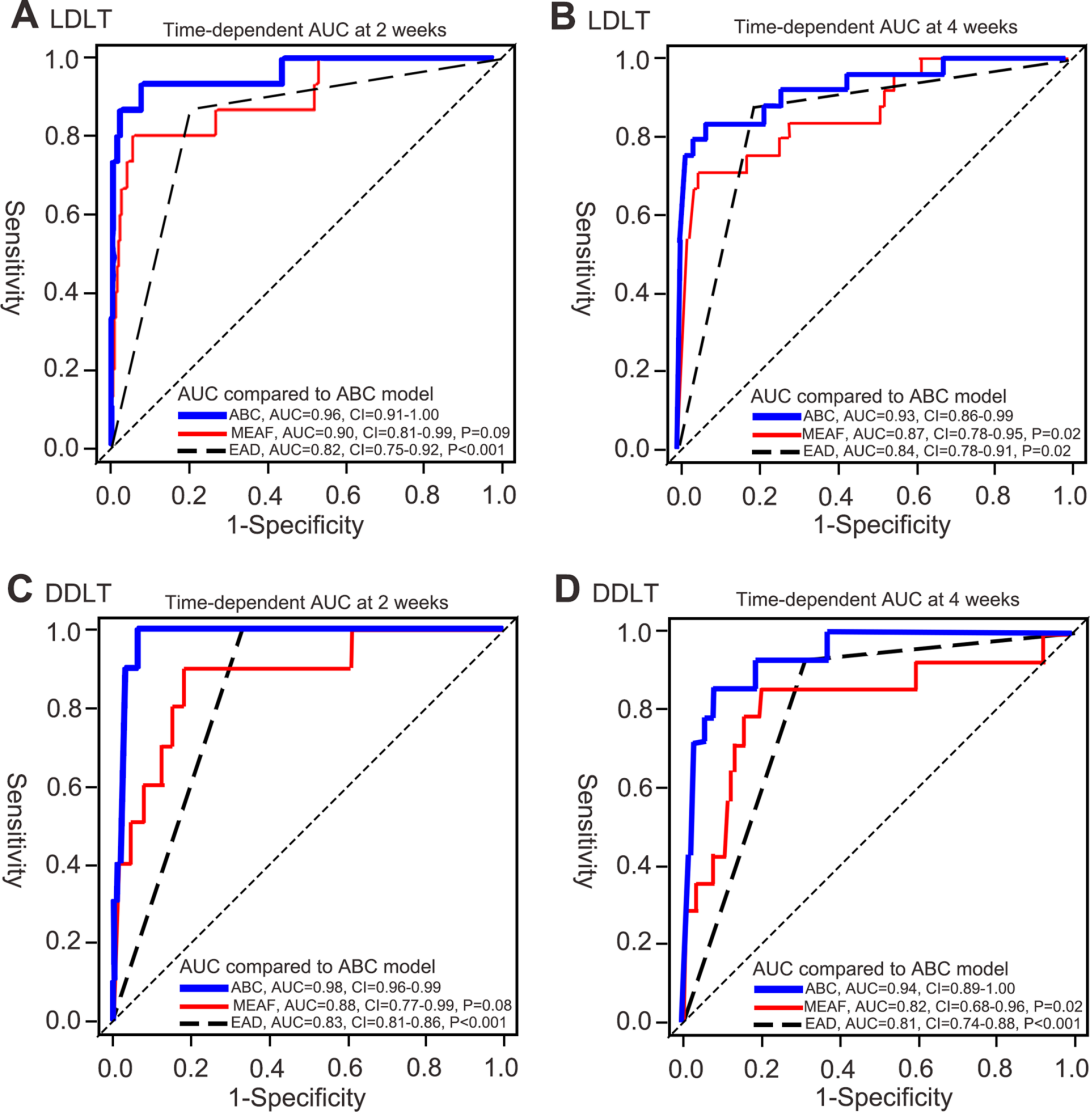
Comparison of time-dependent AUCs of ABC model, MEAF score and EAD criteria. (**A**) Time-dependent AUC at 2 weeks for LDLT. (**B**) Time-dependent AUC at 4 weeks for LDLT. (**C**) Time-dependent AUC at 2 weeks for DDLT. (**D**) Time-dependent AUC at 4 weeks for DDLT. The P values presented are comparison of AUC of MEAF score and EAD criteria against that of ABC model.

C-index and time-dependent AUCs at 2 weeks and 4 weeks were compared between the ABC model and the other two models. The C-index of the ABC model for LDLT (0.73, CI = 0.67–0.79) were higher compared to C-indexes of MEAF score (0.69, CI = 0.63–0.76, P = 0.03) and EAD criteria (0.64, CI = 0.59–0.83, P = 0.001). Time-dependent AUC at 2 weeks of the ABC model (AUC = 0.96, CI = 0.91–1.00) was significantly higher compared to that of EAD criteria (AUC = 0.83, CI = 0.75–0.92, P < 0.001), while there was no significant difference compared to that of MEAF score. (AUC = 0.90, CI = 0.81–0.99, P = 0.09, Fig. 1A) Time-dependent AUC at 4 weeks of the ABC model (AUC = 0.93, CU = 0.86–0.99) was significantly higher compared to those of MEAF score (AUC = 0.87, CI = 0.78–0.95, P = 0.02) and EAD criteria (AUC = 0.84, CI = 0.78–0.91, P = 0.02, Fig. 1B).

Table 3 summarizes the results of the Cox regression models for DDLT. MEAF score as a continuous variable was significantly related to graft survival (HR = 1.65, CI = 1.32–2.06, P < 0.001). EAD criteria as a binary variable was significantly related to graft survival (HR = 2.99, CI = 1.66–5.37, P < 0.001). In the univariate analysis, log_2_-transformed AST_max7_ (HR = 1.57, CI = 1.30–1.90, P < 0.001), log_2_-transformed TB_max3–7_ (HR = 1.70, CI = 1.37–2.10, P < 0.001) and log_2_-transformed INR_max3–7_ (HR = 5.56, CI = 3.46–8.94, P < 0.001) were related to graft survival. The ABC model for DDLT were constructed using three variables; log_2_-transformed AST_max7_ (HR = 1.19, CI = 0.96–1.47, P < 0.11), log_2_-transformed TB_max3–7_ (HR = 1.35, CI = 1.05–1.73, P = 0.02) and log_2_-transformed INR_max3–7_ (HR = 3.07, CI = 1.67–5.64, P < 0.001).

**Table 3:** Comparisons of C-index, time-dependent AUC at 2 weeks, and time-dependent AUC at 4 weeks between Cox proportional hazard regression models using MEAF score, EAD criteria and newly developed multivariable model for predicting graft survival of recipients who underwent deceased donor liver transplantation.

Models	Variables	HR	95% CI	P	C-index	95% CI	P value vs. model	Time-dependent AUC at 2 weeks	95% CI	P value vs. model	Time-dependent AUC at4 weeks	95% CI	P value vs. model
MEAF score		1.65	1.32–2.06	< 0.001	0.71	0.62–0.80	0.31	0.88	0.77–0.99	0.08	0.82	0.68–0.96	0.02
EAD criteria		2.99	1.66–5.37	< 0.001	0.66	0.59–0.73	0.002	0.83	0.81–0.86	< 0.001	0.81	0.74–0.88	< 0.001
Univariable	Log_2_(AST_max7_)	1.57	1.30–1.90	< 0.001									
	Log_2_(TB_max3–7_)	1.70	1.37–2.10	< 0.001									
	Log_2_(INR_max3–7_)	5.56	3.46–8.94	< 0.001									
Multivariable model	Log_2_(AST_max7_)	1.19	0.96–1.47	0.11									
	Log_2_(TB_max3–7_)	1.35	1.05–1.73	0.02	0.74	0.65–0.83		0.98	0.96–1.00		0.94	0.89–1.00	
	Log_2_(INR_max3–7_)	3.07	1.67–5.64	< 0.001									

*HR* hazard ratio, *AUC* area under the curve, *CI* confidence interval, *MEAF* modeling early allograft function, *EAD* early allograft dysfunction, *AST* aspartate aminotransferase, *TB* total bilirubin; *INR* international normalized ratio.

C-index and time-dependent AUCs at 2 weeks and 4 weeks were compared between the ABC model and the other two models. The C-index of the ABC model for DDLT (0.74, CI = 0.65–0.83) was higher compared to C-index of EAD criteria (0.66, CI = 0.59–0.73, P = 0.002) whereas difference with MEAF score was statistically insignificant (0.71, CI = 0.62–0.80, P = 0.31). Time-dependent AUC at 2 weeks of the ABC model (AUC = 0.98, CI = 0.96–0.99) was significantly higher compared to that of EAD criteria (AUC = 0.83, CI = 0.81–0.86, P < 0.001), while there was no significant difference compared to that of MEAF score. (AUC = 0.88, CI = 0.77–0.99, P = 0.08, Fig. 1C) Time-dependent AUC at 4 weeks of the ABC model (AUC = 0.94, CU = 0.89–1.00) was significantly higher compared to those of MEAF score (AUC = 0.82, CI = 0.68–0.96, P = 0.02) and EAD criteria (AUC = 0.81, CI = 0.74–0.88, P < 0.001, Fig. 1D).

The predicted survival probabilities from Cox proportional hazards model for a set of covariates X may be estimated by the equation below where *S*_0_(*t*) is Breslow estimator for baseline survival function.

$$S\left( {t,{\text{ }}x} \right) = S_{0} \left( t \right)^{{exp(x\beta )}}$$

*S*_0_(*t*)—baseline survival function

$$\begin{aligned} x\beta & = \{ (log_{2} AST_{{max7}} \times 0.4205) + (log_{2} TB_{{max3 - 7}} \times 0.3656) + (log_{2} INR_{{max3 - 7}} \times 1.1893)\} - 5.11\quad for\quad LDLT \\ x\beta & = \{ (log_{2} AST_{{max7}} \times 0.1751) + (log_{2} TB_{{max3 - 7}} \times 0.2986) + (log_{2} INR_{{max3 - 7}} \times 1.1205)\} - 3.28\quad for\quad DDLT \\ \end{aligned}$$

The baseline survival function is presented as tables in Supplementary table 1 and 2. The predicted probability of the recipient in a certain time point or the survival plot using the ABC model can be performed by putting the laboratory values into the Excel document provided as Supplementary material.

### Apparent validation and internal validation

For internal validation, 20-time repeated fivefold cross validation was applied to evaluate their performance. Apparent validation of the ABC model for LDLT showed C-index of 0.73 (CI = 0.69–0.79), AUC at 2 weeks of 0.96 (CI = 0.91–1.00), and AUC at 4 weeks of 0.93 (CI = 0.86–0.99). Internal validation using 20-time repeated fivefold cross validation of the ABC model for LDLT showed C-index of 0.68 (CI = 0.66–0.69), AUC at 2 weeks of 0.91 (CI = 0.87–0.96), and AUC at 4 weeks of 0.92 (CI = 0.91–0.94). Apparent validation of the ABC model for DDLT showed C-index of 0.74 (CI = 0.65–0.83), AUC at 2 weeks of 0.98 (CI = 0.96–1.00), and AUC at 4 weeks of 0.94 (CI = 0.89–1.00). Internal validation using 20-time repeated fivefold cross validation of the ABC model for DDLT showed C-index of 0.68 (CI = 0.66–0.70), AUC at 2 weeks of 0.86 (CI = 0.80–0.92), and AUC at 4 weeks of 0.91 (CI = 0.87–0.94).

### Calibration plot

**Figure 2:**
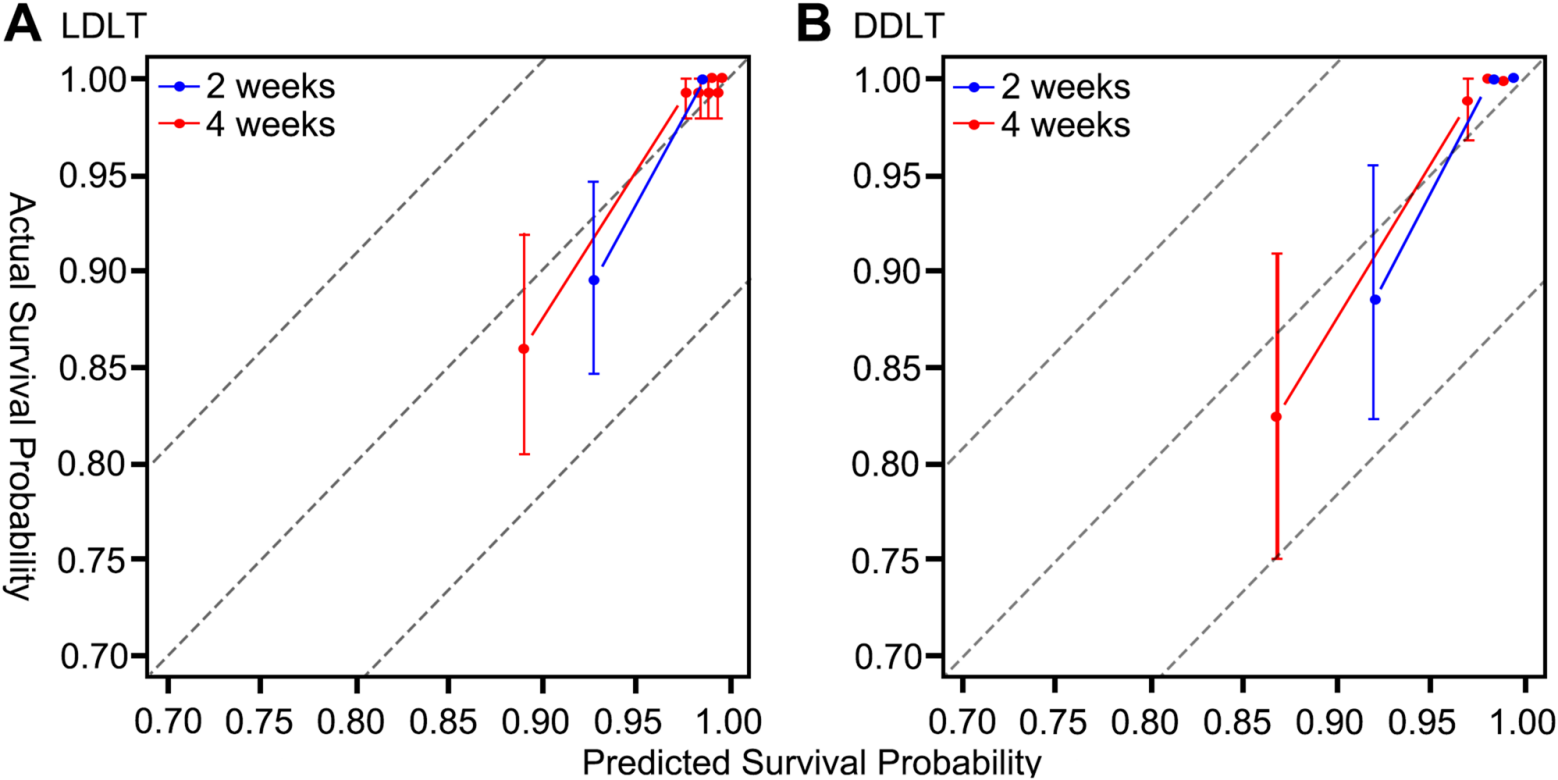
Calibration plots of ABC models for predicting graft survival within 2 weeks and 4 weeks (**A**) in LDLT cases (**B**) and DDLT cases.

Calibration plots of ABC models at 2 weeks and 4 weeks through 1000 bootstrap resamples were performed. Figure 2 shows the calibration plots of ABC models for both LDLT and DDLT. The predicted probability and actual survival probability showed relatively competent calibration for ABC models for LDLT and DDLT.

### Decision curve analysis

**Figure 3:**
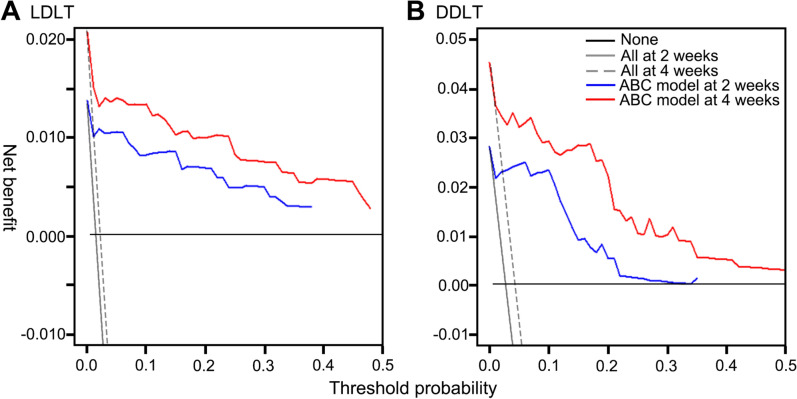
Decision curves of ABC models for predicting graft survival within 2 weeks and 4 weeks (**A**) in LDLT cases (**B**) and DDLT cases.

To evaluate the clinical usefulness of ABC model, decision curves were computed to calculate the net benefit. Figure 3 shows the decision curves of ABC models for LDLT and DDLT. For both 2 weeks and 4 weeks, and for both LDLT and DDLT, the decision curve constantly calculated above the zero-benefit line, showing beneficial expectation of the models.

### Time-dependent AUC curves of ABC model

**Figure 4:**
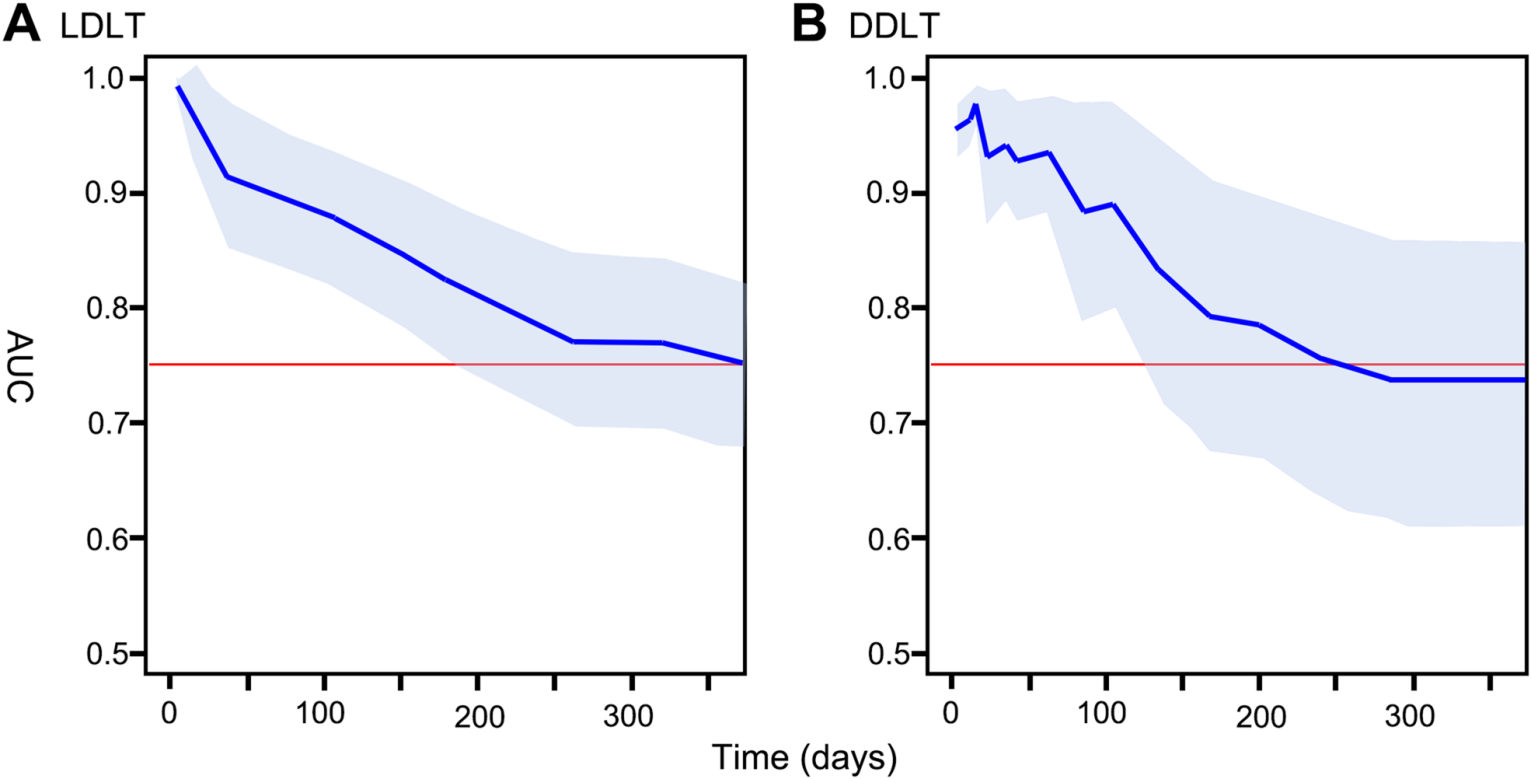
Time-dependent AUC curves during the 1-year post-transplantation period (**A**) in LDLT cases (**B**) and DDLT cases.

Time-dependent AUC curves of ABC models were illustrated in Fig. 4. When the reference line was set as AUC of 0.75, the time-dependent AUCs were calculated to be above the reference line until 1 year in LDLT, and around 250 days in DDLT.

## Discussion

Due to improvement in surgical skills, optimization of immunosuppression, and postoperative intensive care, the outcome of LT has improved throughout the decades, and graft failure rate has significantly decreased. However, there are still recipients who experience graft dysfunction and require appropriate decision making to undergo re-transplantation. Nevertheless, new competent liver grafts for those experiencing graft dysfunction are not always available, creating an urgent need for re-transplant criteria. The criteria of OPTN are utilized as guidance in allocating deceased donor livers although they are limited in allocating new grafts for patients with potential graft failure. Several studies have built a prediction model for graft failure. Although such studies showed improvement in prediction, there is no consensus on a definite model for predicting graft failure. This study was designed to build a prediction model for graft survival using simplified variables among the largest studied cohort.

Nonfunctioning livers usually show a similar pattern of laboratory values. AST and ALT peak at day 1 and 2 post-LT, respectively, and gradually decrease thereafter; there can be additional peaks when the graft is injured by mechanisms such as hypotension. The pattern is similar in successful grafts, but maximum AST and ALT indicate extent of graft injury. Since AST and ALT show similar trend during graft dysfunction, we decided to include only one to the model. On the other hand, TB level changes slowly and gradually increases along the clinical course in failing grafts. The initial TB level is dependent on pre-LT TB level and transfusions, which are performed intensely during the initial post-LT period. Therefore, both successful and failing LTs show a decreasing pattern in the initial period, while failing LTs then show gradual increase. Patterns of INR level are most similar between successful and failing grafts, although the levels are higher in nonfunctioning grafts and remain higher during the post-LT course. However, the time point and level of the peak may vary among LT cases. Therefore, peak AST after LT and maximum TB and INR after the early post-LT period are important regardless of day. This is why we built a model to choose the maximum AST of the post-LT period and maximum values of TB and INR starting from day 3 post-LT.

ABC model was built based on LT data from 1153 LDLT and 359 DDLTs. The reason why separate analyses were performed for LDLT and DDLT was due to the different clinical characteristics. While LDLT uses partial graft with less ischemic injury compared to DDLT, DDLT usually uses whole graft with considerable amount of ischemic injury. The laboratory values after LT are also different between LDLT and DDLT as presented in Table 1. AST, TB and PT/INR of DDLT are higher compared to LDLT in the initial period. As a result, the AUCs of the prediction models were 0.96 and 0.98 in predicting graft failure within 2 weeks and 0.93 and 0.94 in predicting graft failure within 4 weeks, for LDLT and DDLT, respectively. ABC model is also very intuitive by including the maximum values of AST, TB, and INR during the first week for predicting early graft failure. The model was compared to previously published models, such as MEAF score and EAD criteria. By comparing the C-index and time-dependent AUC at 2 weeks and 4 weeks, ABC model showed superior outcome compared to the other two models. The difference of ABC model from other models is that it is optimized for both LDLT and DDLT. While EAD criteria and MEAF score were modeled based on DDLT, our model consists of two versions using same variables. Prediction probability can be calculated easily if the clinician knows the maximum AST, TB, and INR during the post-LT period, by inserting the values to our supplementary Excel document, which is well-calibrated to the retrospective cohort of our institution. Our prediction calculator not only predicts the probability of graft failure at a certain time point after LT but also the graft survival curve which can give visual information useful both for the clinicians and patients.

The limitation of our study is that it is based on data from a single institution. The model was based on a cohort of predominantly LDLT and number of cases included in the DDLT model was 359 cases. EAD criteria and MEAF score were based on DDLT cases which is more dominantly performed worldwide. The EAD criteria has been extensively validated while ABC model is only on the starting point. Nevertheless, our study showed high validity during internal validation; therefore, good results during external validation with other cohorts is expected. The two different models with same statistical approach is also the strength of our study. Although many countries are performing LT with DDLT, there are still many countries with significant number of LDLT. ABC model will serve as a good tool for predicting early graft failure after LDLT.

Whether it is advantageous to use ABC model instead of traditional measures is up to the clinicians. While we showed that the statistical data showed superior outcome of ABC model compared to the two models, some clinicians might consider that the two traditional measures are more useful since they also showed good statistical outcome and were validated by other investigators. Our model was based on single institutional data consisted of Korean patients which is expected to be different to cohorts used for other models. Therefore, we propose other investigators to externally validate ABC model.

The currently applied criteria for primary nonfunction as suggested by OPTN served as a good decision tool. However, the criteria were quite restrictive; in countries like the Republic of Korea where donation from deceased donors is relatively lower than in other countries, many patients with graft failure are unable to undergo re-LT with liver from deceased donor. Our prediction model provides objective data on the probability of graft survival, which can guide patient selection in those requiring urgent re-LT even the first week after LT. For the future, ABC model should be validated by other cohort.

## Supplementary Information

Supplementary Tables.

## Abbreviations

LT: Liver transplantation
OPTN: Organ Procurement and Transplant Network
AST: Aspartate aminotransferase
INR: International normalized ratio
TB: Total bilirubin
EAD: Early allograft dysfunction
MEAF: Modeling early allograft function
LDLT: Living donor liver transplantation
DDLT: Deceased donor liver transplantation
AUC: Area-under-the-curve
ROC: Receiver-operating-characteristics
CI: Confidence interval
IQR: Interquartile range
OR: Hazard ratio
