# Supplementary material for: Prediction model for early graft failure after liver transplantation using aspartate aminotransferase, total bilirubin and coagulation factor
Source: Sci Rep. 2021 Jun 18;11:12909. doi: 10.1038/s41598-021-92298-6 (PMC8213713; doi:10.1038/s41598-021-92298-6)
Supplement: Supplementary file 1 — Supplementary Tables. [file 41598_2021_92298_MOESM1_ESM.docx]

Supplementary table 1. Baseline survival function of ABC model for LDLT.

| FU_dur_day | S_0_(t) |
| --- | --- |
| 0 | 1 |
| 3 | 0.9996 |
| 4 | 0.9992 |
| 5 | 0.9984 |
| 6 | 0.9971 |
| 7 | 0.9967 |
| 8 | 0.9949 |
| 9 | 0.9944 |
| 11 | 0.9934 |
| 14 | 0.9928 |
| 15 | 0.9918 |
| 16 | 0.9907 |
| 17 | 0.9901 |
| 20 | 0.9895 |
| 21 | 0.9890 |
| 22 | 0.9884 |
| 31 | 0.9878 |
| 34 | 0.9872 |
| 36 | 0.9866 |
| 37 | 0.9860 |
| 40 | 0.9854 |
| 41 | 0.9847 |
| 47 | 0.9841 |
| 57 | 0.9835 |
| 64 | 0.9828 |
| 68 | 0.9821 |
| 80 | 0.9808 |
| 82 | 0.9801 |
| 104 | 0.9794 |
| 123 | 0.9787 |
| 126 | 0.9780 |
| 127 | 0.9773 |
| 134 | 0.9766 |
| 147 | 0.9758 |
| 150 | 0.9751 |
| 154 | 0.9744 |
| 164 | 0.9737 |
| 171 | 0.9730 |
| 186 | 0.9722 |
| 190 | 0.9715 |
| 202 | 0.9708 |
| 204 | 0.9700 |
| 227 | 0.9693 |
| 254 | 0.9686 |
| 262 | 0.9678 |
| 294 | 0.9671 |
| 324 | 0.9663 |
| 326 | 0.9656 |
| 364 | 0.9648 |
| 373 | 0.9640 |
| 401 | 0.9632 |
| 412 | 0.9624 |
| 452 | 0.9616 |
| 515 | 0.9607 |
| 534 | 0.9599 |
| 570 | 0.9591 |
| 624 | 0.9582 |
| 662 | 0.9573 |
| 905 | 0.9564 |
| 919 | 0.9555 |
| 953 | 0.9545 |
| 968 | 0.9536 |
| 1320 | 0.9526 |
| 1405 | 0.9515 |
| 1442 | 0.9505 |
| 1556 | 0.9494 |
| 1584 | 0.9483 |
| 1751 | 0.9471 |
| 2204 | 0.9458 |
| 2292 | 0.9443 |
| 2688 | 0.9427 |
| 3369 | 0.9406 |
| 3524 | 0.9380 |
| 3566 | 0.9354 |
| 4080 | 0.9305 |
| 4274 | 0.9252 |

Supplementary table 2. Baseline survival function of ABC model for DDLT.

| Day after LT | S_0_(t) |
| --- | --- |
| 0 | 1 |
| 2 | 0.9985 |
| 5 | 0.9970 |
| 9 | 0.9938 |
| 10 | 0.9906 |
| 11 | 0.9873 |
| 13 | 0.9837 |
| 18 | 0.9797 |
| 21 | 0.9777 |
| 24 | 0.9757 |
| 28 | 0.9713 |
| 35 | 0.9691 |
| 41 | 0.9669 |
| 42 | 0.9645 |
| 56 | 0.9622 |
| 62 | 0.9597 |
| 72 | 0.9571 |
| 89 | 0.9546 |
| 107 | 0.9519 |
| 134 | 0.9492 |
| 135 | 0.9466 |
| 151 | 0.9439 |
| 198 | 0.9411 |
| 221 | 0.9382 |
| 288 | 0.9352 |
| 370 | 0.9321 |
| 463 | 0.9286 |
| 524 | 0.9250 |
| 631 | 0.9211 |
| 704 | 0.9172 |
| 757 | 0.9132 |
| 887 | 0.9089 |
| 1076 | 0.9044 |
| 1402 | 0.8993 |
| 2210 | 0.8923 |
| 2532 | 0.8838 |
| 2825 | 0.8745 |
| 2939 | 0.8651 |
| 3010 | 0.8557 |
| 3149 | 0.8461 |
| 3325 | 0.8358 |
